# Residual Compressive Strength of Short Tubular Steel Columns with Artificially Fabricated Local Corrosion Damage

**DOI:** 10.3390/ma13040813

**Published:** 2020-02-11

**Authors:** Kyra Kamille Toledo, Hyoung-Seok Kim, Young-Soo Jeong, In-Tae Kim

**Affiliations:** 1 School of Urban, Architecture and Civil Engineering, Pusan National University, Busan 46241, Korea; toledokyra@gmail.com (K.K.T.); knet7654@naver.com (H.-S.K.); 2 Seismic Testing Center, Pusan National University, Yangsan 50612, Korea; ysjung@pusan.ac.kr

**Keywords:** short tubular steel column, residual compressive strength, finite element analysis, local corrosion, corroded volume, half-wavelength of buckling

## Abstract

Corrosion is considered as one of the main factors in the structural performance deterioration of steel members. In this study, experimental and numerical methods were used to assess the reduction in compressive strength of short tubular steel columns with artificially fabricated local corrosion damage. The corrosion damage was varied with different depths, heights, circumferences, and locations along the column. A parametric numerical study was performed to establish a correlation between the residual compressive strength and the severity of corrosion damage. The results showed that as the corrosion depth, height and circumference increased, the compressive strength decreased linearly. As for the corrosion height, the residual compressive strength became constant after decreasing linearly when the corrosion height was greater than the half-wavelength of buckling of the short columns. An equation is presented to evaluate the residual compressive strength of short columns with local corrosion wherein the volume of the corrosion damage was used as a reduction factor in calculating the compressive strength. The percentage error using the presented equation was found to be within 11.4%.

## 1. Introduction

Corrosion is one of the main contributors in the decrease of the structural performance of steel structures and develops in various outdoor conditions, such as rural, urban, and marine environments. Due to the low drag resistance and ease of handling during construction of tubular members, it is generally used in offshore structures as structural elements [1,2,3]. Corrosion causes reduction in the cross-sectional area of steel tubular members, making it prone to local buckling, and may lead to compressive failure [4,5,6]. Therefore, it is important to examine the reduction in compressive strength of short tubular steel columns that are subjected to corrosion damage.

Hebor et al. [1] and Nazari et al. [2] used a numerical approach to evaluate the residual compressive strength of locally corroded steel tubular members in offshore structures by considering the depth and circumference of the corrosion. They also suggested equations to predict the residual strength of tubes with patch corrosion. Oyaester et al. [6] and Lutes et al. [7] proposed equations to predict the buckling capacity of a tubular specimen according to the corrosion depth. Ahn et al. [8] studied the effect of a marine environment on steel members and conducted an experimental analysis on circular tubular columns with inclined corrosion damage. They also recommended an equation to evaluate the residual compressive strength of a tubular member with local corrosion. Zeinoddini et al. [9] evaluated the effect of corrosion to steel tubular members with exact as-is morphology acquisition using numerical approaches.

**Figure 1:**
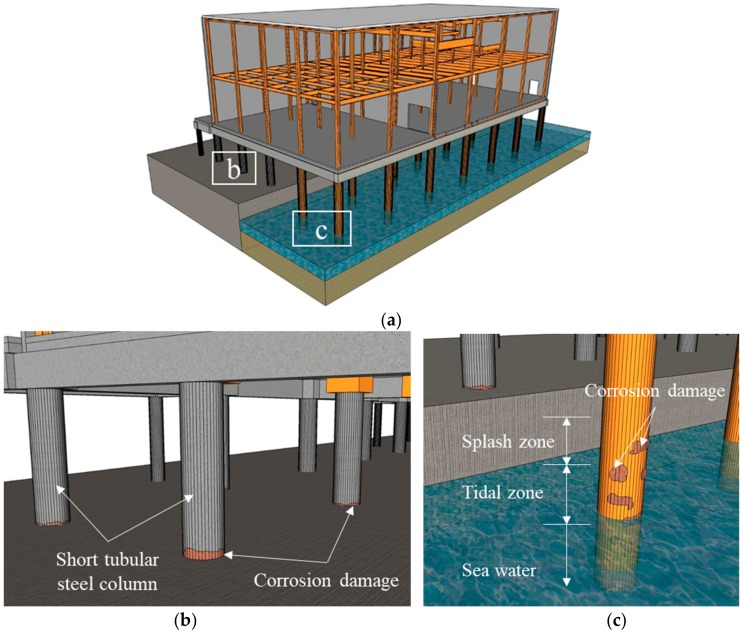

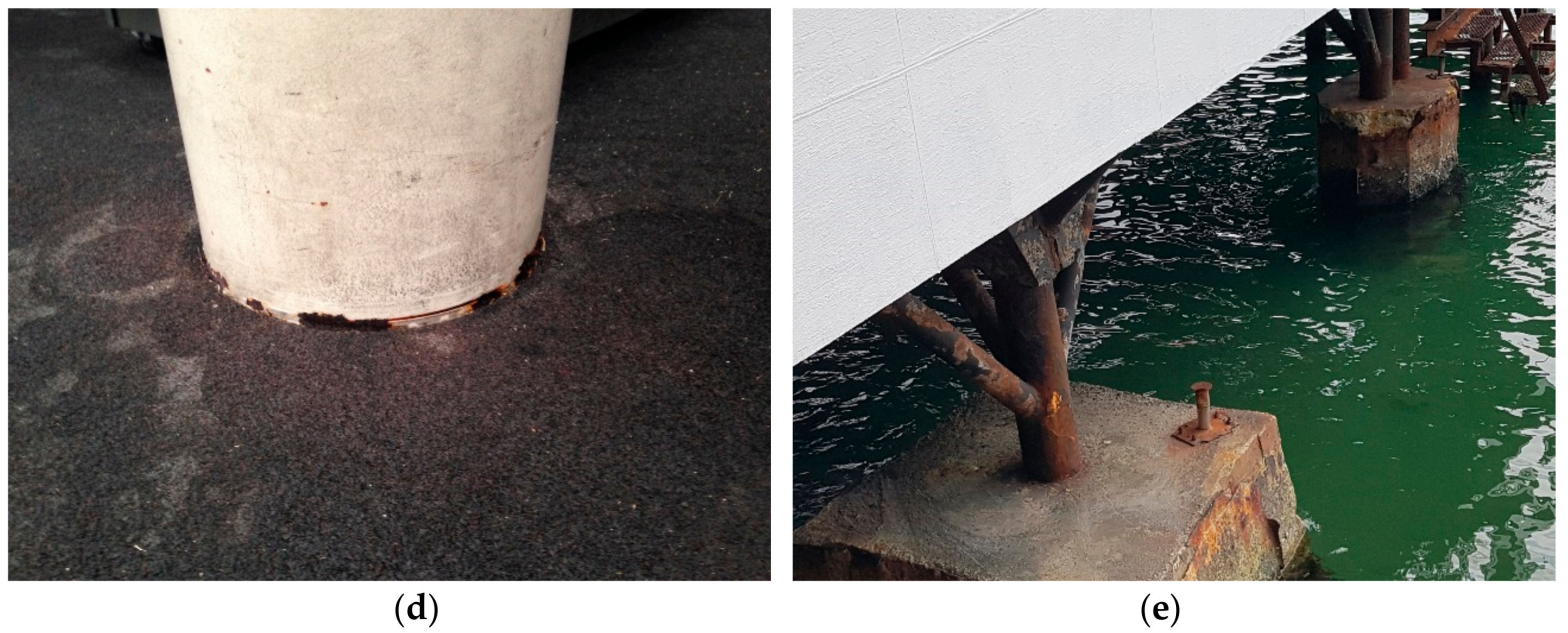
Occurrence of corrosion in short tubular steel columns: (**a**) Different exposure environments of an offshore structure; (**b**) Corrosion at ends; (**c**) Corrosion along the column; (**d**) Actual corrosion at ends; (**e**) Actual corrosion along the column.

Previous studies have conceptualized corrosion damage as a two-dimensional body where the depth and circumference of the corrosion are only considered [1,2,10]. The corrosion height may be a critical factor in the compressive strength of columns that it is ideal to consider it together with corrosion depth and circumference. Furthermore, the corrosion damage is usually located at the middle of the columns that it is necessary to analyze the effect of damage at different locations along the column caused by different exposure environments of the columns as shown in Figure 1a. Figure 1b,d show that local corrosion frequently occurs at the ends because dust and moisture can easily accumulate there and accelerates the local or uniform corrosion of the short tubular steel column. Additionally, the exposure condition and coating deterioration are important to be considered as factors in the corrosion propagation of the columns as shown in Figure 1c,e.

Experimental compressive tests and finite element modeling were used to evaluate the residual compressive strength of short tubular steel columns subjected to local corrosion. The damage of the short columns differs in the depth, height, circumference, and location of corrosion along the column. Then, a parametric finite element study was also carried out to establish a relationship between the residual compressive strength and the corrosion depth, height and circumference of the short columns. Moreover, a simple calculation approach was presented to evaluate the residual compressive strength by using the volume of the corrosion damage as a reduction factor.

## 2. Experimental and Numerical Analysis

### 2.1. Experimental Study

#### 2.1.1. Test Specimen

The test specimens were made of STPG 370 structural steel pipe that complies with the ASTM A500 standard [11] and were fabricated into short tubular steel columns with local corrosion at the ends. The uniform corrosion thickness was artificially fabricated using a milling machine. The thickness of the specimen was checked and obtained a maximum percentage error of 2.5% between the designed and manufactured thickness. The manufactured thickness was used to evaluate the residual compressive strength in this paper.

**Figure 2:**
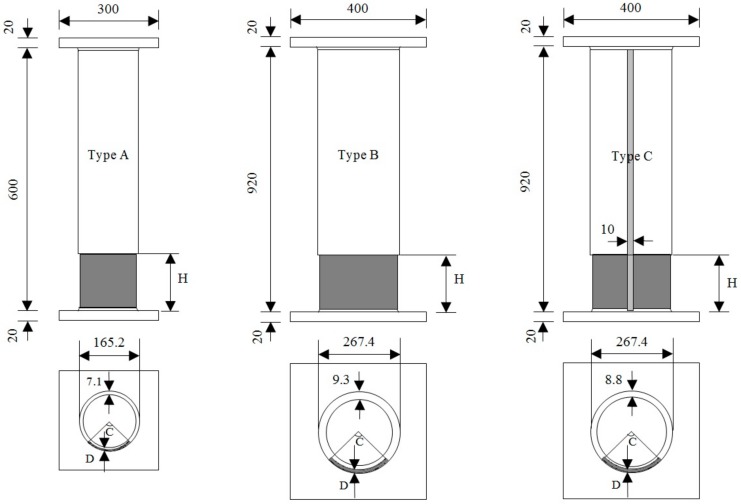
Dimensions of Type A, Type B, and Type C short tubular steel columns (units: mm).

**Figure 3:**
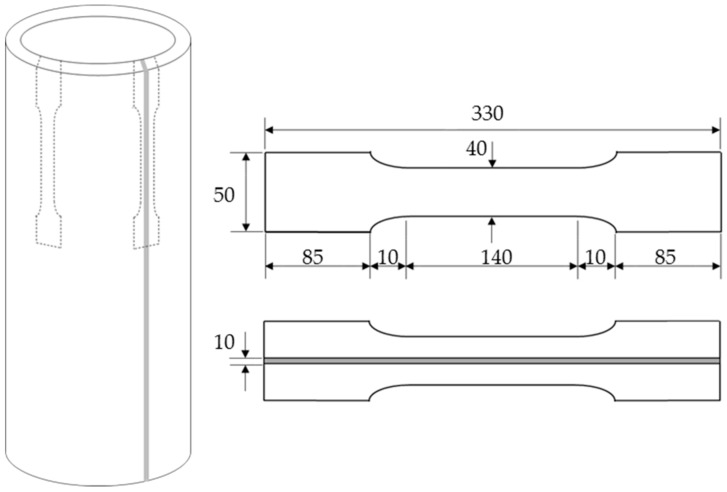
Tensile test specimen (units: mm).

Three types of test specimens named Types A, B and C were analyzed in the experimental study and are shown in Figure 2 [4,5]. Types A and B are both seamless columns where Type A has a smaller scale than B. Type A columns have an external diameter of 165.2 mm, wall thickness of 7.1 mm, and column length of 600 mm. Type B columns have an external diameter of 267.4 mm, wall thickness of 9.3 mm, and column length of 920 mm. Moreover, Types B and C have the same dimensions and their difference is that the Type C columns are welded with a weld thickness of 10 mm, as shown in Figure 2. A total of 21 short columns were experimentally tested. The material properties and chemical composition of the columns are presented in Table 1. A tensile test was conducted to check the material properties of the steel used in the fabrication of the columns. The tensile test specimens are shown in Figure 3 and collected from each type of column, where applicable.

The local corrosion pattern in the columns varied in depth (0, 1.5, 2, 3, 4, 4.5, and 6 mm), height (0, 20, 40, 60, 80, 100, 120, 140, 160, and 180 mm), and circumference (0, 90, 180, 270, and 360°), as presented in Table 2. Designations were used to identify each specimen based on the depth (D), height (H), circumference (C), and type of short tubular steel column. Hence, D3H20-C360A is a Type A column with D = 3 mm, H = 20 mm, and C = 360°. As another example, D0H0-C0A is a Type A column which has zero corrosion depth, height, and circumference and is considered as a reference column with no corrosion.

#### 2.1.2. Compressive Test

The short tubular steel columns were placed on a universal testing machine (UTM) of 5000 kN capacity for the compressive tests and loaded to failure. A displacement-controlled method was used with loading rates of 1 mm/min and 5 mm/min before and after the yield point, respectively [12]. The yield loads were then noted after the compressive tests.

### 2.2. Numerical Analysis

#### 2.2.1. Finite Element Analysis of Experimental and Supplementary Specimen

Finite element analysis was carried out using the software SIMULIA ABAQUS 6.14 to perform the simulations of the same short tubular steel columns tested to failure in the laboratory. Material properties, presented in Table 1, from the tensile tests were used in the finite element models. There were 21 models with element type C3D8R used in the numerical analysis. Reduced integration model elements were used in the finite element software to consider large strains on the specimen [13]. Moreover, it usually provides more accurate results and significantly reduces running time, especially in three-dimensional analysis [13].

Boundary conditions were applied to the ends of the columns. All degrees of freedom of the lower end are constrained, while the upper end of the column was fully restrained except on its longitudinal or axial axis. A displacement-controlled method was used for loading to assess the post-buckling behavior of the column. The total compression in the model is divided into a number of steps in which the shape and axial shortening of the specimen are calculated based on the previous steps [13]. The deformation at the end of each step is considered as the starting deformation for the next step [13].

**Figure 4:**
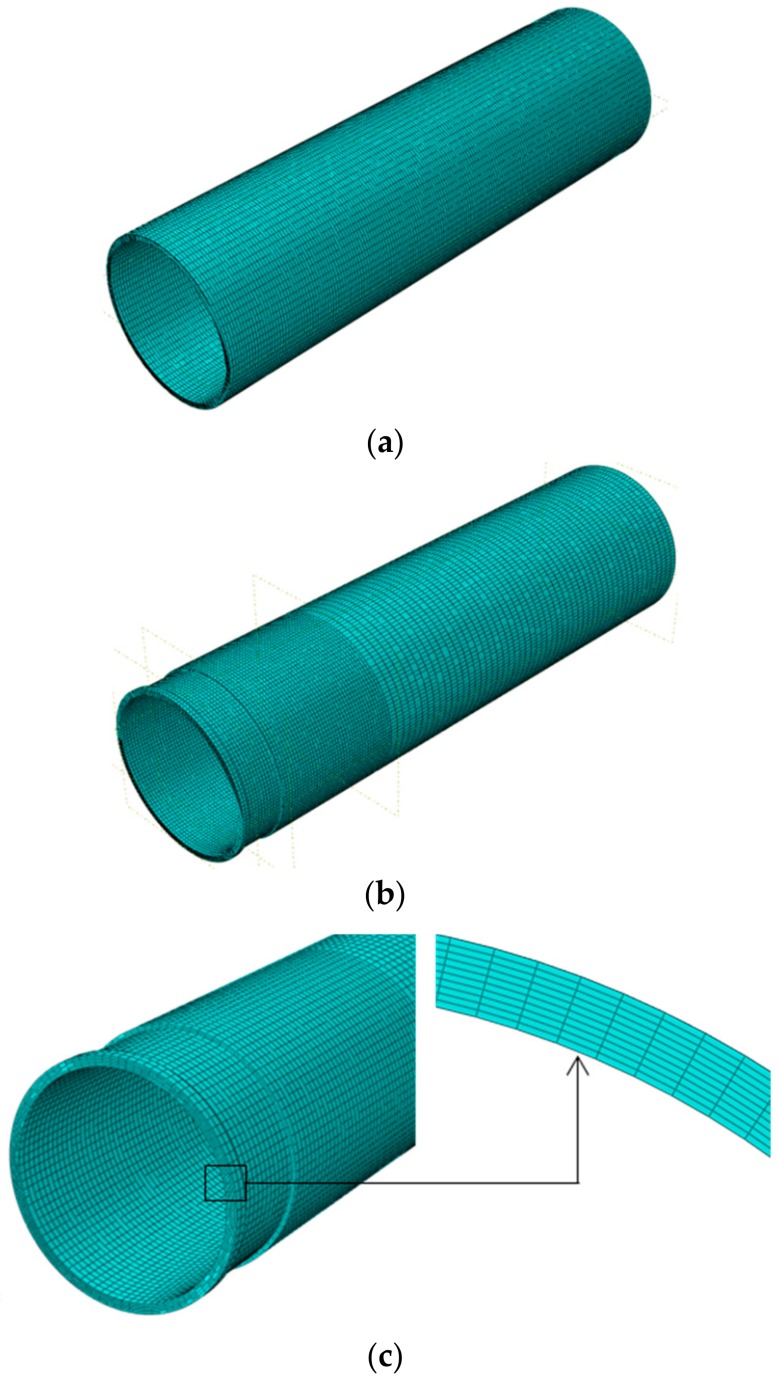
A sample of a finite element model mesh: (**a**) Non-corroded model; (**b**) Corroded model; (**c**) Transverse axis mesh.

Regular meshing was established for non-corroded specimens, and then irregular meshing was considered for the corroded specimens. As shown in Figure 4, a coarser mesh was applied to areas where peculiar deformations were not observed and a fine mesh was applied to the corroded areas with size of 5 mm and 1 mm along the longitudinal and transverse axis, respectively. A nonlinear analysis was performed through the Static Riks Analysis step in the software, which was used to plot the load capacity-vertical displacement curves. The yield load was considered as the residual compressive strength of the columns because the yield stress is associated with the allowable stress of a tubular column according to AISI specifications [14].

Moreover, a parametric study was conducted to establish a relationship between the residual compressive strength of the columns and the corrosion geometry. A total of 18 additional short tubular steel columns with varied corrosion depth (2, 3, 4 and 8 mm), height (20, 40, 60, 80, 100, 110, 140 and 160 mm) and circumference (90°, 180° and 270°) were modeled in ABAQUS 6.14 and the characteristics of the specimens are presented in Table 3.

#### 2.2.2. Finite Element Analysis Considering Varied Corrosion Location

**Figure 5:**
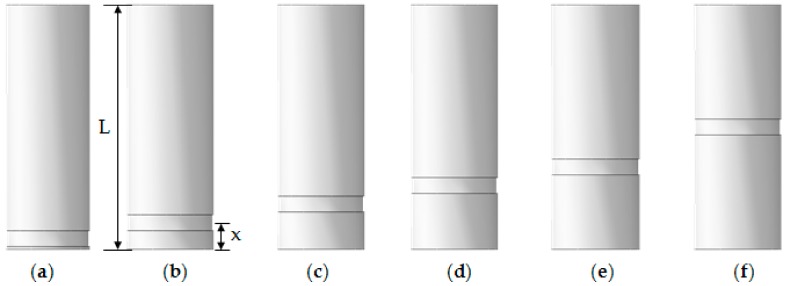
Corrosion damage location in terms of the distance between the bottom of the column and the middle height of corroded area, *x*/*L*: (**a**) 0.0; (**b**) 0.1; (**c**) 0.2; (**d**) 0.3; (**e**) 0.4; (**f**) 0.5.

It is necessary to analyze change in compressive behavior with various locations of corrosion damage since corrosion may occur at different sections along a column depending on its environment. An addition of five short tubular steel columns was modeled with varied locations to consider the effect on the residual compressive strength. Due to the symmetry of the column geometry, the locations of the corrosion damage were varied from the end towards the middle of the column, as shown in Figure 5. The locations of the corrosion are presented in terms of *x*/*L*, where *L* stands for the total length of columns and x for the distance between the bottom of column and the middle height of corroded area.

## 3. Experimental and Numerical Analysis Results

### 3.1. Experimental Results

**Figure 6:**
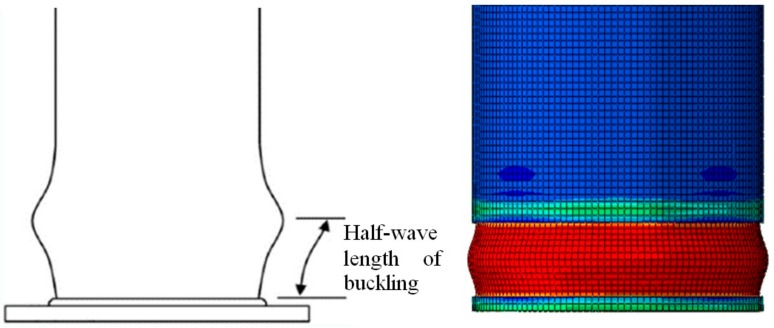
Length of half-wave buckling of a short tubular steel column.

When the short tubular columns are subjected to axial compression, the columns experienced local buckling that resembled an elephant-foot in the corroded areas. The symmetrical columns were observed to have uniform buckling during the tests. The asymmetrical columns have non-uniform buckling on one side due to the variable wall thickness but after a certain time during loading, the other side of the column buckled to support the load and the column’s buckling became approximately symmetrical. A sample of the buckling is shown in Figure 6. The half-wavelength of buckling was used to analyze the behavior of the short columns. According to Timoshenko et al. [15], the half-wavelength is defined as the distance between the bottom of the column and the maximum lateral displacement exhibited during axial compression. The half-wavelength *H_e_* for a non-corroded short column is given in the following Equation (1) [15]:

(1) $$H_{e}=1.72\sqrt{Rt},$$

where *R* is the radius of the column, and *t* is the thickness of the wall.

**Figure 7:**
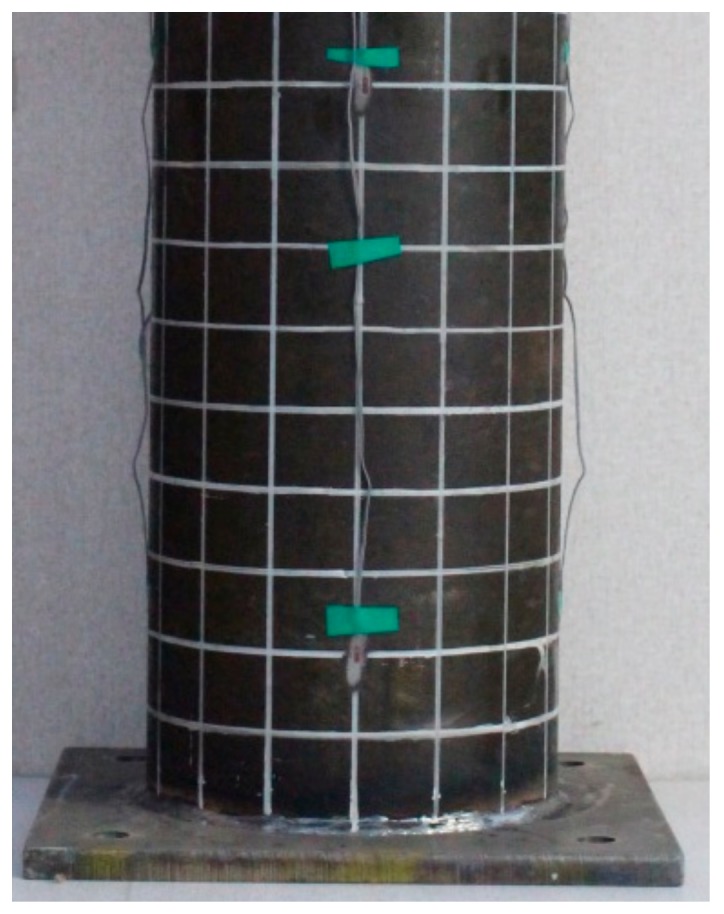
Drawn grid of 5 cm intervals.

The calculated half-wavelength of buckling for Types A, B, and C were 40 mm, 60 mm, and 60 mm, respectively, which were approximately equal to the measured values from the experimental specimen. The half-wavelength of buckling was measured by a drawn grid on the columns with 5 cm intervals as shown in Figure 7.

**Figure 8:**
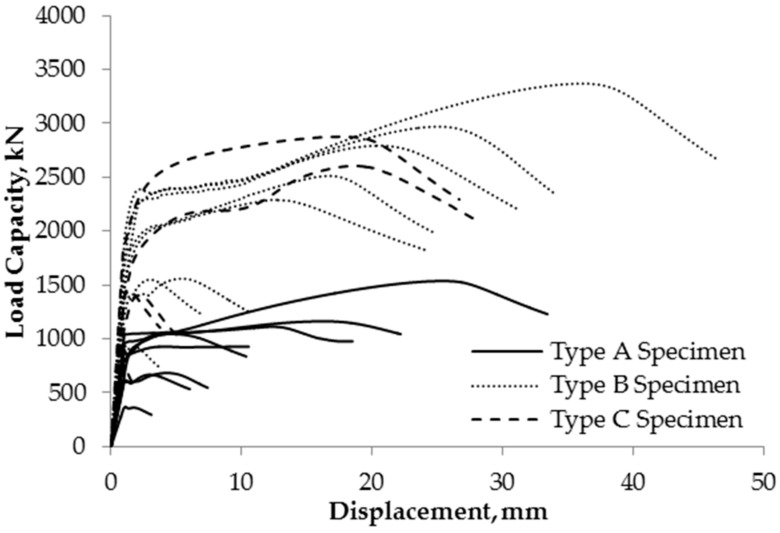
Experimental load-displacement curves.

**Figure 9:**
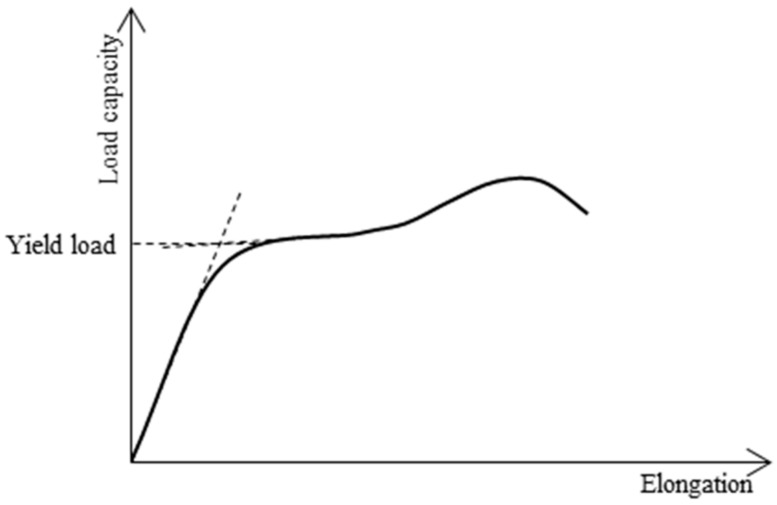
Coplan’s yield point method.

The load capacity versus vertical displacement curves were plotted using the results of the experimental compressive tests as shown in Figure 8. When the yield load is not distinct, the Coplan’s yield point method [16] shown in Figure 9 was utilized to determine the yield load of the specimen. The resulting experimental yield loads, P_EXP_, are summarized in Table 4 and it shows that the columns with corrosion have lower compressive strength compared to the non-corroded columns.

### 3.2. Numerical Analysis Results

**Figure 10:**
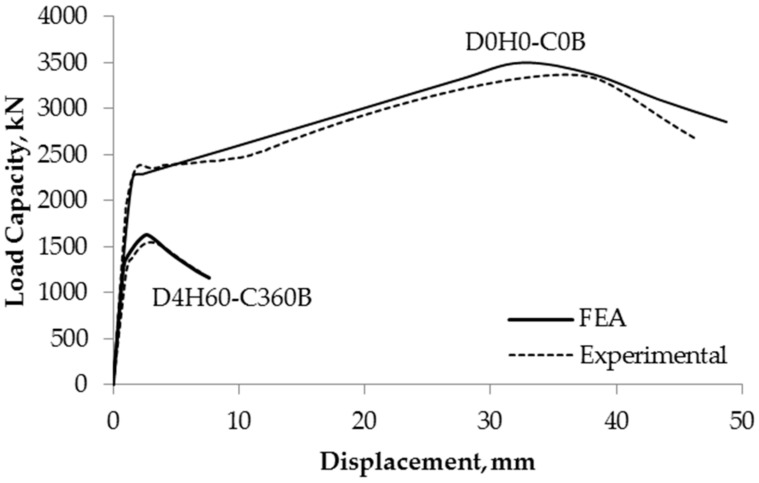
Comparison of experimental results and numerical results of non-corroded and corroded specimens.

Finite element analysis results were plotted into load capacity versus vertical displacement curves. The numerical analysis yield loads, P_FEA_, were summarized in Table 4. The loads were determined using the plotted load-displacement curves, and if the yield load is not distinct, the Coplan’s yield point method [16] was used. A sample of the load-displacement curves for specimens D0H0-C360B (non-corroded) and D4H60-C360B (corroded) are shown in Figure 10. It is evident that a significant loss of capacity can occur when a column has local corrosion.

### 3.3. Supplementary Analysis Results

**Figure 11:**
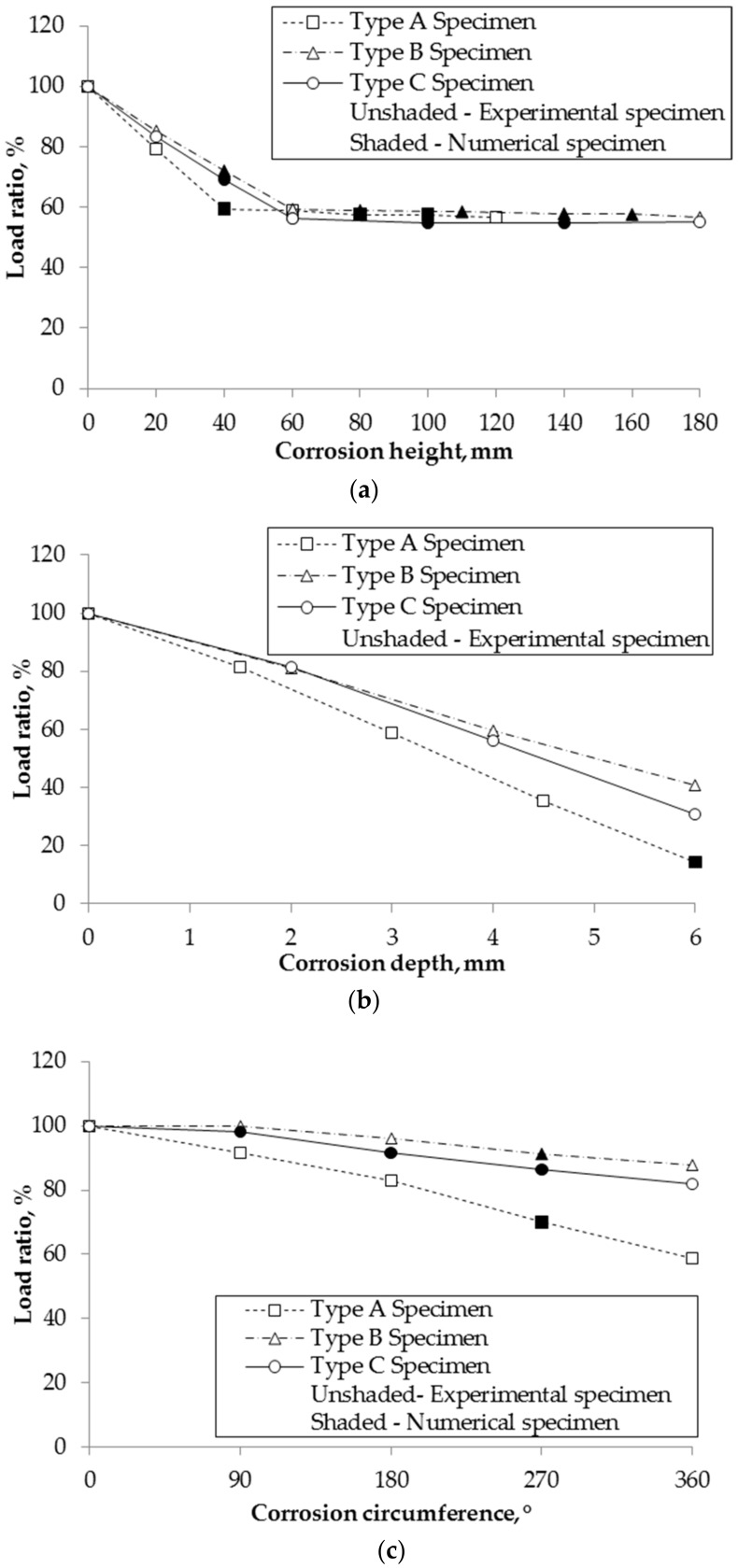
Relationship of residual compressive strength and corrosion level: (**a**) Load ratio with respect to corrosion height; (**b**) Load ratio with respect to corrosion depth; (**c**) Load ratio with respect to corrosion circumference.

A parametric study was done using finite element modeling to analyze the relationship between the compressive behavior of short tubular steel columns and corrosion depth, height and circumference. In order to confirm the impact of each corrosion parameter on yield load ratio, the relationships between each corrosion parameter and the load ratios were plotted in Figure 11. The load ratio is the ratio of the yield load of a corroded specimen to that of a non-corroded specimen. Figure 11a shows the load ratio with respect to the specimens of different corrosion heights with the same corrosion depth and circumference. The residual compressive strength starts to become constant at corrosion heights of 40 mm, 60 mm, and 60 mm for Types A, B and C, respectively. These values are also identical to the half-wavelength of buckling (Equation (1)) of the columns. When corrosion height is shorter than half-wavelength of buckling, it affects the strength and buckling behavior of column. This leads to the change in yield location and yield load. In contrast, when corrosion height is higher than the half-wavelength, buckling occurs at the same location and the strength does not change any longer. Therefore, the load ratio linearly decreases while the corrosion height increases and it becomes constant when the corrosion height is more than the half-wavelength of column.

Figure 11b illustrates the relationship between the load ratios and specimens of different corrosion depths with the same corrosion height and circumference. The compressive strength decreases linearly as the corrosion depth increases. Figure 11c shows the load ratio with respect to the specimens of different corrosion circumferences while having the same corrosion depth and height, and the capacity decreases linearly as the corrosion circumference increases.

A comparison between the results of the experiment and the numerical analysis is shown in Table 4. This is to confirm the reliability of finite element models to be used in the parametric studies. The maximum percentage error between the experimental and numerical analysis is 2.40% for Type A columns, 8.90% for Type B columns, and 5.38% for Type C columns. It is observed that there is greater loss of load capacity for deeper, longer, and wider corrosion area.

The percentage error was within 10% in a parametric study which was done to support the relationship between the residual compressive strength of the columns and the corrosion geometry, and to analyze the effect of varied corrosion damage location along the short columns.

**Figure 12:**
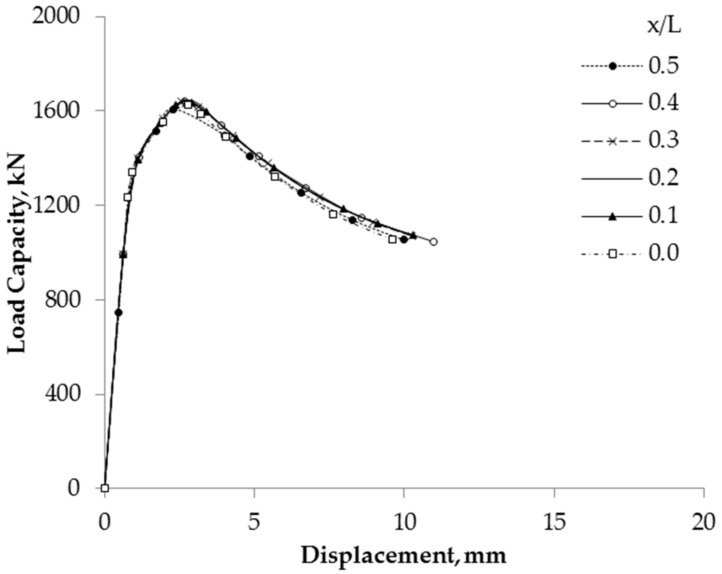
Compressive behavior of specimen D4H60-C360B depending on different locations.

Furthermore, the effect of the different locations of the corrosion damage on residual compressive strength was also considered. The results revealed that the residual compressive strength is independent of the location of corrosion damage since the load-displacement curves are approximately the same for the different locations, as shown in Figure 12.

### 3.4. Present Equation to Evaluate Residual Compressive Strength

The compressive strength of an axially loaded short tubular steel column without corrosion damage can be evaluated by Equation (2) [7]. From the experimental and numerical results, it can be identified that reduction of compressive strength is a function of the corrosion depth, height and circumference. The reduction factor in the residual compressive strength of the short columns is the ratio of the residual volume with respect to the non-corroded volume, as shown in Equation (3). Equation (3) is formulated by taking the ratio of corrosion depth, height and circumference with respect to the wall thickness and half-wavelength of buckling of the short column.

(2) $$P_{y}=2\pi Rt\sigma_{y},$$

where *P_y_* is the yield load of the short column without corrosion damage, *R* is the radius of the column, *t* is the initial wall thickness and *σ_y_* is the yield stress.

(3) $$\frac{V_{residual}}{V_{non-corroded}}=1-\frac{V_{corroded}}{V_{non-corroded}}=1-\frac{d}{t}\frac{H}{H_{e}}\frac{\theta }{360},$$

where $V_{residual}$ is the residual volume, $V_{non-corroded}$ is the non-corroded volume of the column, $V_{corroded}$ is the corroded volume of the column, d is the corrosion depth, H is the corrosion height, and $H_{e}$ is the half-wavelength of buckling. $H_{e}$ is used in the equation when H is greater than $H_{e}$. Finally, θ is the corrosion circumference (°).

Then, Equation (2) is combined with Equation (3) to evaluate the residual compressive strength of a short tubular steel column with local corrosion damage (Equation (4)).

(4) $$P_{y-corroded}=2\pi Rt\sigma_{y}\left(\frac{V_{residual}}{V_{non-corroded}}\right),$$

where $P_{y-corroded}$ is the residual compressive yield load of the columns. The calculated compressive yield loads are presented in Table 5.

The percentage error between the experimental results and calculated values using Equation (4) was within 6.8% for Type A columns, 11.4% for Type B columns, and 8.6% for Type C columns. When using Equation (4) to evaluate the residual compressive strength, a maximum percentage error between the experimental and calculated results was 11.4%.

### 3.5. Comparison of Present and Previous Equations

Previous researchers, Hebor et al. [1] and Nazari et al. [2], have conducted experimental, numerical studies and proposed equations to evaluate the residual compressive strength of short tubular steel members with patch corrosion at varied locations along the column. The corrosion depth and circumference were the considered parameters for the specimens since the corrosion height was set to a constant value greater than the half-wavelength of buckling for all columns. The corroded specimen was artificially fabricated by mechanically removing the material with the use of a hand-held electric powered grinder. Then, finite element analysis using the experimental parameters was carried out to compare with the experimental results. From the results, Hebor et al. [1] recommended an equation that relates the residual compressive strength of a locally corroded tube to its patch corrosion dimensions:

(5) $$\frac{P_{y-Hebor}}{P_{y}}=0.052\left(\frac{t_{r}}{t}\right)-0.001\left(\frac{D}{t}\right)0.0026\left(\theta \right)+0.0028\left(\theta \right)\left(\frac{t_{r}}{t}\right)+0.9998$$

Whereas Nazari et al. [2] improved Hebor’s equation to estimate the residual compressive strength of tubular members:

(6) $$\frac{P_{y-Nazari}}{P_{y}}=0.02\left(\frac{t_{r}}{t}\right)-0.001\left(\frac{D}{t}\right)-0.007\left(\frac{L}{D}\right)-0.0022\theta +0.0018\theta \left(\frac{t_{r}}{t}\right)+1.0415$$

where $P_{y-Hebor/Nazari}$ is the compressive yield load of corroded columns, $P_{y}$ is the yield load of the short column without corrosion damage, $t_{r}$ is the residual wall thickness, t is the initial wall thickness, L is the column length, D is the outside diameter of the column, and θ is the angle of corrosion damage in degrees. The established equations were used for specimens having the following parameters: $0\leq \frac{t_{r}}{t}\leq 1,\;22\leq \frac{D}{t}\leq 100\;\mathrm{and}\;15{}^{\circ}\leq \theta \leq 360{}^{\circ}$. The design parameters of the short columns in this study conform to the conditions of the previous equations.

These equations are applicable to only a specific range of short tubular members and were applied to short columns that have patch corrosion. Equations (5) and (6) were used for comparison to the present equation in this study. The calculated compressive yield loads are shown in Table 5.

Comparing the values between the experimental results and calculated values using Equation (5), the percentage errors were 2.8 to 26.4% for Type A columns, 1.6 to 31.0% for Type B specimens and 1.0 to 28.1% for Type C columns. Whereas the percentage errors between experimental yield loads and calculated yield loads from Equation (6) were 3.6 to 31.2% for Type A specimens, 4.6 to 35.6% for Type B specimens and 1.4 to 33.4% for Type C specimens.

It can be observed that the columns D3H20-C360A, D4H20-C360B, and D4H20-C360C possessed the highest percentage error when using the equations of Hebor et al. [1] and Nazari et al. [2]. These columns have the same corrosion height of 20 mm, which is less than the half-wavelength of buckling. It shows that Equation (5) and Equation (6) cannot be applied to such columns with corrosion height of less than the value of the half-wavelength since Nazari et al. [1] and Hebor et al. [2] used a fixed corrosion height that is greater than the half-wavelength of buckling.

**Figure 13:**
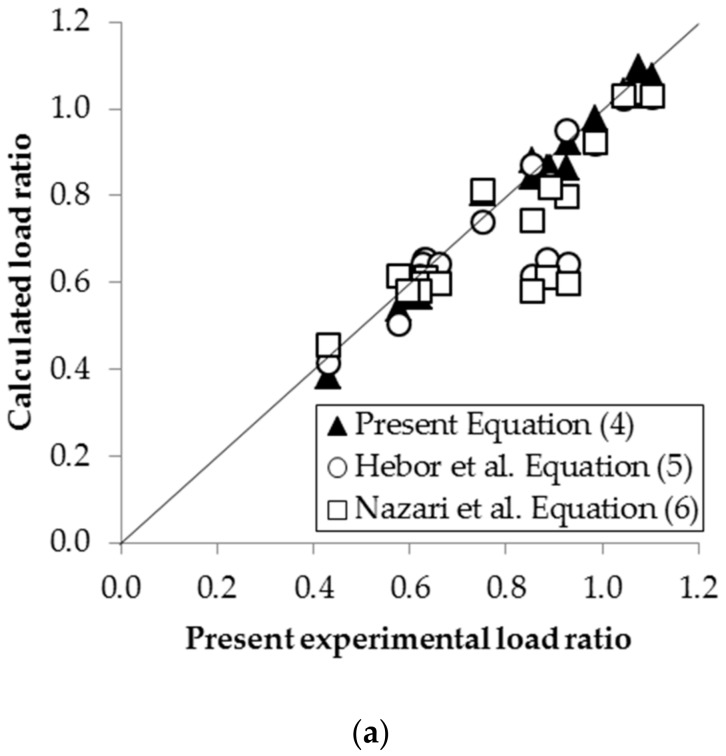

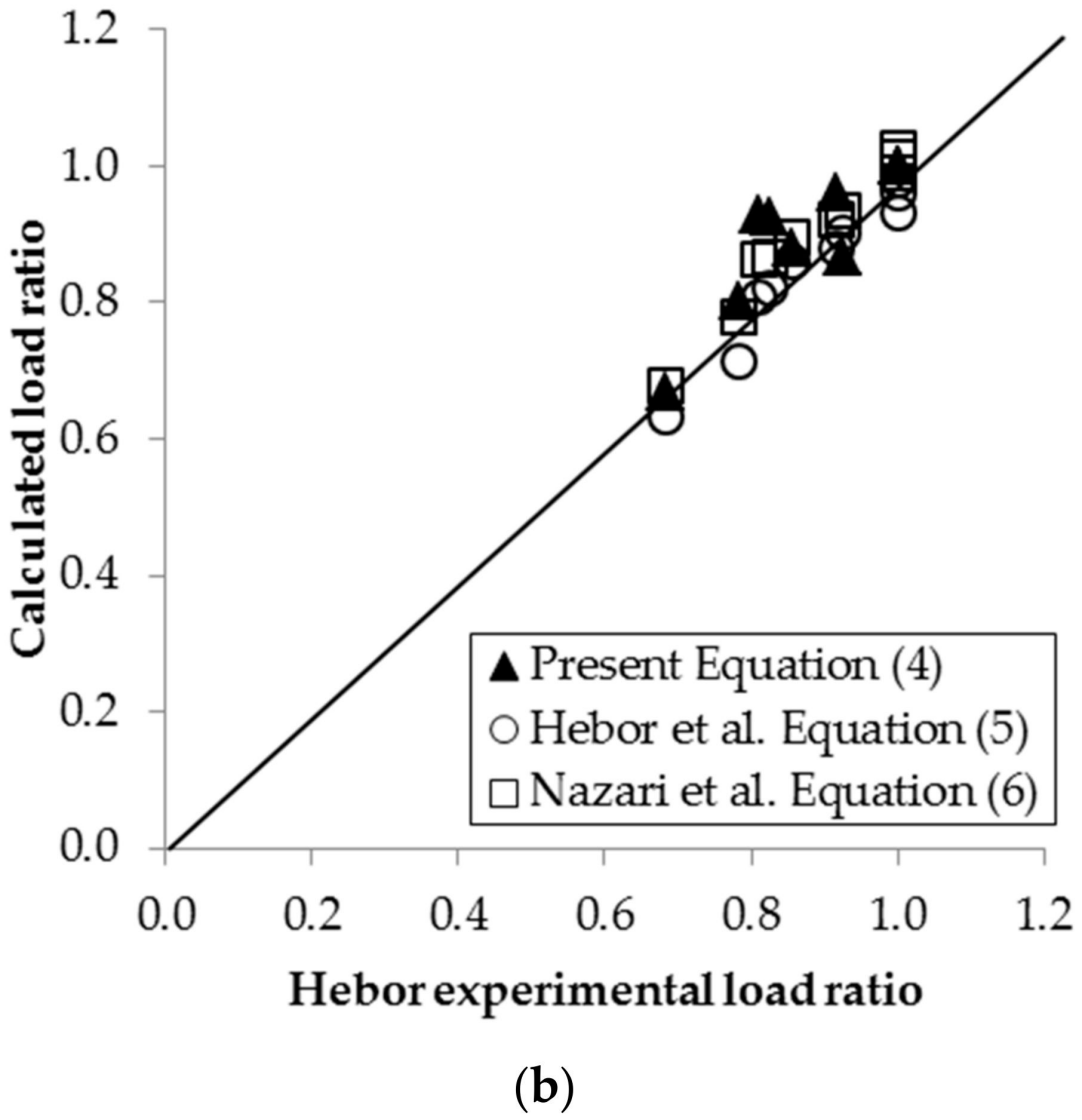
Comparison of experimental and calculated residual compressive strength: (**a**) Comparison of present experimental results versus calculated load ratio; (**b**) Hebor’s [1] experimental results versus calculated load ratio.

The experimental load ratios and calculated load ratios are compared in Figure 13a. The results showed that the residual compressive strength of short tubular steel columns can be evaluated using the present equation, Equation (4), within a maximum percentage error of 11.4%. In addition, the experimental results of Hebor et al. [1] and the calculated load ratios are compared in Figure 13b. It was found that applying Equation (4) to obtain the residual compressive strength resulted in a maximum error of 12.3%.

## 4. Conclusions

This study used experimental and numerical methods to evaluate the reduction in compressive strength of short tubular steel columns with artificially fabricated local corrosion damage. Different depths, heights, circumferences, and locations of local corrosion were studied, and the following results were found:

The severity of corrosion has great effects on the reduction of the compressive strength of the short tubular steel columns. The half-wavelength of buckling is also a critical factor for the residual compressive strength of steel tubular short columns. The residual compressive strength decreases linearly with the corrosion height up to a point where it becomes constant. The change in the trend is associated with the half-wavelength of buckling. The residual compressive strength is independent of the corrosion height if it is greater than the half-wavelength of buckling dimension. Furthermore, as the corrosion depth and circumference increase, the residual compressive strength shows a linear decreasing trend.

The same load capacity versus vertical displacement curve was obtained when corrosion damage was variedly located along the short column since it has the same corroded volume. The residual compressive strength of a short tubular steel column is independent of the location of corrosion damage.

A simple approach was presented to evaluate the residual compressive strength of a column with local corrosion and made use of the residual volume of corrosion damage:

(7) $$P_{y-corroded}=2\pi Rt\sigma_{y}\left(\frac{V_{residual}}{V_{non-corroded}}\right)$$

A maximum error of 11.4% can be obtained when using this equation to evaluate the residual compressive strength of such columns.

## Figures and Tables

**Table 1:** Material properties and chemical composition of STPG 370.

Specimen	Material Properties		Chemical Composition (wt%)				
	Yield Stress	Tensile Stress	C	Si	Mn	P	S
	(MPa)	(MPa)					
Type A	288	465	0.230	0.200	0.690	0.020	0.008
Type B	298	489	0.220	0.230	0.690	0.018	0.007
Type C	342 (Parent metal)	416 (Parent metal)	0.150	0.100	0.390	0.014	0.007
	419 (Weld metal)	475 (Weld metal)					

**Table 2:** Corrosion data of experimental specimen.

**Type A Specimen**		**Corrosion Depth–Corrosion Circumference**			
		**0 mm**	**1.5 mm**	**3 mm**	**4.5 mm**
Corrosion Height	0 mm	D0H0-C0A			
	20 mm			D3H20-C360A	
	60 mm		D1H60-C360A	D3H60-C360A	D4H60-C360A
				D3H60-C180A	
				D3H60-C90A	
	120 mm			D3H120-C360A	
**Type B Specimen**		**Corrosion Depth–Corrosion Circumference**			
		**0 mm**	**2 mm**	**4 mm**	**6 mm**
Corrosion Height	0 mm	D0H0-C0B			
	20 mm			D4H20-C360B	
				D4H20-C180B	
				D4H20-C90B	
	60 mm		D2H60-C360B	D4H60-C360B	D6H60-C360B
	180 mm			D4H180-C360B	
**Type C Specimen**		**Corrosion Depth–Corrosion Circumference**			
		**0 mm**	**4 mm**	**6 mm**	
Corrosion Height	0 mm	D0H0-C0C			
	20 mm		D4H20-C360C		
	60 mm		D4H60-C360C	D6H60-C360C	
	180 mm		D4H180-C360C		

**Table 3:** Corrosion data of supplementary specimen.

**Type A Specimen**		**Corrosion Depth–Corrosion Circumference**	
		**3 mm**	**6 mm**
**Corrosion Height**	40 mm	D3H40-C360A	
	60 mm	D3H60-C270A	
	80 mm	D3H80-C360A	D6H80-C360A
	100mm	D3H100-C360A	
**Type B Specimen**		**Corrosion Depth–Corrosion Circumference**	
		**4 mm**	**8 mm**
Corrosion Height	20 mm	D4H20-C270B	
	40 mm	D4H40-C360B	
	60 mm		D8H60-C360A
	80 mm	D4H80-C360B	
	110 mm	D4H110-C360B	
	140 mm	D4H140-C360B	
	160 mm	D4H160-C360B	
**Type C Specimen**		**Corrosion Depth–Corrosion Circumference**	
		**2 mm**	**4 mm**
Corrosion Height	20 mm		D4H20-C90C†D4H20-C180C†D4H20-C270C
	60 mm	D2H60-C360C	
	100 mm		D4H100-C360C
	140 mm		D4H140-C360C

**Table 4:** Comparison of experimental and numerical results.

Specimen	Yield Load (kN)		Percentage Error
	P_EXP_	P_FE__A_	%
D0H0-C0A	1043	1018	2.40
D3H20-C360A	860	840	2.33
D3H60-C360A	613	620	1.14
D3H120-C360A	614	600	2.28
D1H60-C360A	898	880	2.00
D4H60-C360A	368	370	0.54
D3H60-C90A	955	937	1.88
D3H60-C180A	865	850	1.73
D0H0-C0B	2388	2290	4.10
D4H20-C360B	2013	1910	5.12
D4H60-C360B	1360	1350	0.74
D4H180-C360B	1431	1400	2.17
D2H60-C360B	1853	1890	2.00
D6H60-C360B	933	850	8.90
D4H20-C90B	2289	2230	2.58
D4H20-C180B	2200	2190	0.45
D0H0-C0C	2455	2500	1.83
D4H20-C360C	2008	1900	5.38
D4H60-C360C	1458	1400	3.98
D4H180-C360C	1401	1350	3.64
D6H60-C360C	756	775	2.51

**Table 5:** Calculated residual compressive yield load.

Specimen	$P_{EXP}$	$P_{y-corroded}$	$P_{y-Hebor}$	$P_{y-Nazari}$	Expt. vs. Equation (4)	Expt. vs. Equation (5)	Expt. vs. Equation (6)
	(kN)	Equation (4) (kN)	Equation (5) (kN)	Equation (6) (kN)	% Error	% Error	% Error
D0H0-C0A	1043	1061.2	997.7	1005.2	1.7	4.3	3.6
D3H20-C360A	860	837.3	633.1	591.4	2.6	26.4	31.2
D3H60-C360A	613	612.3	633.1	591.4	0.1	3.3	3.5
D3H120-C360A	614	612.3	633.1	591.4	0.3	3.1	3.7
D1H60-C360A	898	837.3	922.7	774.2	6.8	2.8	13.8
D3H60-C90A	955	948.7	890.6	895.3	0.7	6.7	6.2
D3H60-C180A	865	837.3	804.7	794.0	3.2	7.0	8.2
D0H0-C0B	2388	2328.1	2220.5	2232.2	2.5	7.0	6.5
D4H20-C360B	2013	1995.2	1388.8	1297.0	0.9	31.0	35.6
D4H60-C360B	1360	1327.0	1388.8	1297.0	2.4	2.1	4.6
D4H180-C360B	1431	1327.0	1388.8	1297.0	7.3	2.9	9.4
D2H60-C360B	1853	1827.6	1882.6	1608.7	1.4	1.6	13.2
D6H60-C360B	933	826.5	895.0	985.3	11.4	4.1	5.6
D0H0-C0C	2455	2444.0	2407.7	2420.2	0.4	1.9	1.4
D4H20-C360C	2008	2074.0	1443.7	1366.1	3.3	28.1	32.0
D4H60-C360C	1458	1333.0	1443.7	1366.1	8.6	1.0	6.3
D4H180-C360C	1401	1333.0	1443.7	1366.1	4.9	3.0	2.5
